# Cordycepin Induces S Phase Arrest and Apoptosis in Human Gallbladder Cancer Cells

**DOI:** 10.3390/molecules190811350

**Published:** 2014-07-31

**Authors:** Xu-An Wang, Shan-Shan Xiang, Huai-Feng Li, Xiang-Song Wu, Mao-Lan Li, Yi-Jun Shu, Fei Zhang, Yang Cao, Yuan-Yuan Ye, Run-Fa Bao, Hao Weng, Wen-Guang Wu, Jia-Sheng Mu, Yun-Ping Hu, Lin Jiang, Zhu-Jun Tan, Wei Lu, Ping Wang, Ying-Bin Liu

**Affiliations:** 1 Institute of Biliary Tract Disease, Xinhua Hospital, School of Medicine, Shanghai Jiao Tong University, No. 1665 Kongjiang Road, Shanghai 200092, China; 2 Laboratory of General Surgery, Xinhua Hospital, School of Medicine, Shanghai Jiao Tong University, No. 1665 Kongjiang Road, Shanghai 200092, China; 3 Department of General Surgery, School of Medicine, Shanghai Jiao Tong University, No. 1665 Kongjiang Road, Shanghai 200092, China; 4 Department of General Surgery, Hangzhou People’s First Hospital, No. 261 Huansha Road, Hangzhou 310009, China

**Keywords:** cordycepin, gallbladder cancer cells, proliferation, cell cycle, apoptosis

## Abstract

Gallbladder cancer is the most common malignant tumor of the biliary tract, and this condition has a rather dismal prognosis, with an extremely low five-year survival rate. To improve the outcome of unresectable and recurrent gallbladder cancer, it is necessary to develop new effective treatments and drugs. The purpose of the present study was to evaluate the effects of cordycepin on human gallbladder cells and uncover the molecular mechanisms responsible for these effects. The Cell Counting Kit-8 (CCK-8) and colony formation assays revealed that cordycepin affected the viability and proliferation of human gallbladder cancer cells in a dose- and time-dependent manner. Flow cytometric analysis showed that cordycepin induced S phase arrest in human gallbladder cancer cell lines(NOZ and GBC-SD cells). Cordycepin-induced apoptosis was observed using an Annexin V/propidium iodide (PI) double-staining assay, and the mitochondrial membrane potential (ΔΨm) decreased in a dose-dependent manner. Additionally, western blot analysis revealed the upregulation of cleaved-caspase-3, cleaved-caspase-9, cleaved-PARP and Bax and the downregulation of Bcl-2, cyclin A and Cdk-2 in cordycepin-treated cells. Moreover, cordycepin inhibited tumor growth in nude mice bearing NOZ tumors. Our results indicate that this drug may represent an effective treatment for gallbladder carcinoma.

## 1. Introduction

Gallbladder cancer, an aggressive and highly lethal malignancy, is the most frequent cancer of the biliary tract and the most common neoplasm of the digestive system [1,2,3,4,5]. The diagnosis of gallbladder cancer often occurs at an advanced stage due to non-specific signs and presenting symptoms. This late diagnosis results in many gallbladder cancer cases being non-resectable at the time of presentation. As a result, gallbladder cancer has a very poor prognosis, with a five-year survival rate of less than 10%, and the median survival rate for patients with locally advanced gallbladder cancer is approximately 3–6 months [6,7]. Complete resection is the primary curative treatment for gallbladder cancer; unfortunately, more than half of gallbladder cancer patients have unresectable tumors at diagnosis, and recurrence after surgery is common. Palliative therapy, such as chemotherapy and radiotherapy, are often introduced to improve prognosis; however, these treatments are often ineffective [8,9]. To improve the outcome of unresectable and recurrent gallbladder cancer, it is necessary to develop new effective treatments and drugs.

**Figure 1:**
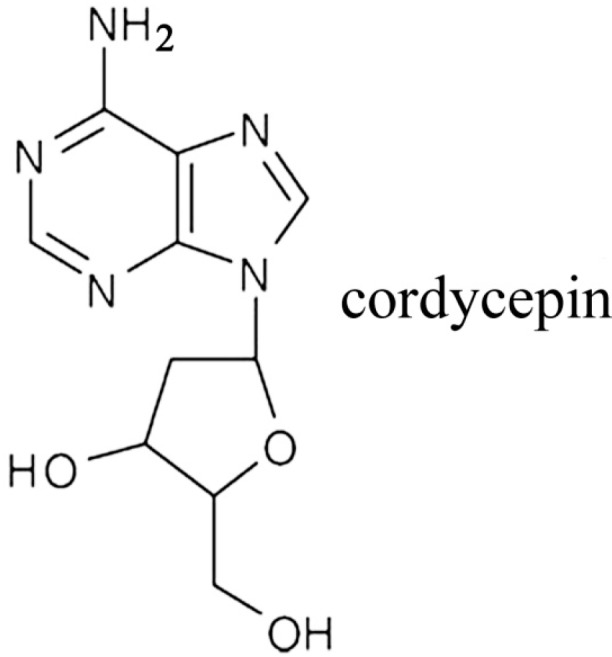
Chemical structure of cordycepin.

Cordycepin (3'-deoxyadenosine) (Figure 1), a chief ingredient and the active component of *Cordyceps sinensis*, is widely used in traditional Chinese medicine [10,11]. This drug is a derivative of the nucleoside, adenosine, but lacks an oxygen in the 3' position of its ribose moiety, which results in the termination of chain elongation during RNA synthesis. The activity of cordycepin has been well described *in vitro* using purified RNA polymerases and poly(A) polymerases from a number of organisms, including yeast and mammals [12]. In clinical trials, cordycepin has been shown to possess a variety of pharmacological properties, such as anti-inflammatory (with overall enhancement of immune function), anti-aging and anticancer effects. These anticancer effects have been observed in oral, lung, bladder, prostate, hepatic and colorectal carcinoma and mainly involve the induction of apoptosis and cell cycle arrest via the targeting of specific molecules and pathways [13,14,15,16,17,18,19]. However, to our knowledge, the effect of cordycepin on gallbladder cancer cells has not been previously investigated. The purpose of the present study was to evaluate the effects of cordycepin on human gallbladder cells and uncover the molecular mechanisms responsible for these effects.

## 2. Results and Discussion

### 2.1. Cordycepin Inhibits Proliferation and Colony Formation of Gallbladder Cancer Cells

**Figure 2:**
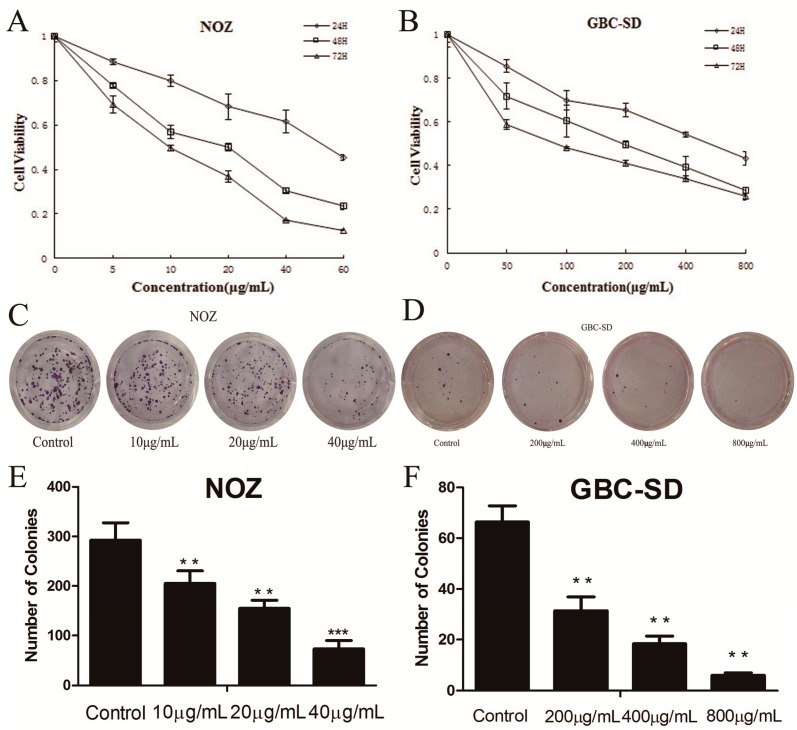
Cordycepin inhibits the proliferation and colony formation of gallbladder cancer cells.

To confirm the inhibitory effect of cordycepin on cell proliferation, the CCK-8 assay was used. After treatment with cordycepin at various concentrations (0, 5, 10, 20, 40 and 60 μg/mL for NOZ cells and 0, 0.05, 0.1, 0.2, 0.4 and 0.8 mg/mL for GBC-SD cells) for 24, 48 and 72 h, both NOZ and GBC-SD cells showed a dose- and time-dependent decrease in viability. Growth curves for these experiments are shown in Figure 2A,B. The IC_50_ (the concentration at which 50% inhibition of cell growth was achieved) of cordycepin in NOZ and GBC-SD cells at 48 h was approximately 19.2 μg/mL and 398.1 μg/mL, respectively, which indicates that cordycepin could inhibit the proliferation capability of gallbladder cancer cells.

Additionally, we investigated the effect of cordycepin on the proliferation of gallbladder cancer cells using a colony assay. As shown in Figure 2C,D, the number of colonies of cordycepin-treated GBC-SD and NOZ cells was significantly lower than that in the control group (Figure 2E,F). These data show that cordycepin has an anti-proliferative effect on gallbladder cancer cells.

In the experiment, we found that the effective concentration of cordycepin was much lower for NOZ cells. The main conclusion to draw from this observation is that not all cancer cell lines are equally sensitive to cordycepin. This makes it likely that not all gallbladder tumors will respond to cordycepin, and it is therefore important for the development of cordycepin as a drug to understand what determines cordycepin sensitivity, so that the correct patient group to treat with the drug can be identified. Additionally, we guess that this may be caused by differences in AMP-activated protein kinase (AMPK) expression in these gallbladder cancer cells, and we are planning to perform western blots assay for AMPK gamma isoforms to confirm it. AMPK is an energy-sensing enzyme that maintains the balance between ATP production and consumption in all eukaryotic cells. AMPK can sense cellular energy levels; when the energy status is compromised, the system activates catabolic pathways and switches off protein, carbohydrate and lipid biosynthesis, as well as cell growth and proliferation [20,21].

(**A**,**B**) NOZ and GBC-SD cells were treated with various concentrations of cordycepin for 24, 48 and 72 h. Cell viability was assessed using the CCK-8 assay. (**C**,**F**) Cordycepin suppressed colony formation of GBC-SD and NOZ cells. Cells were treated with cordycepin and then cultured in fresh medium for 14 days to form colonies. Values represent the mean ± SD of three independent experiments. ** *p* < 0.01, *** *p* < 0.001.

### 2.2. Cordycepin Induces S Phase Arrest and Regulates the Expression of Cell Cycle-Related Proteins in Gallbladder Cancer Cells

**Figure 3:**
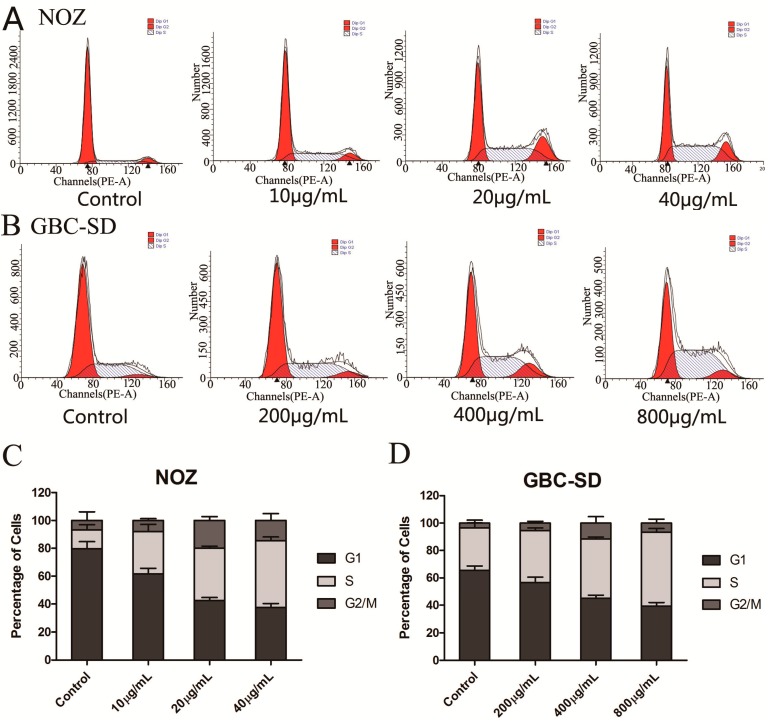
Cordycepin induces S phase arrest and regulates the expression of cell cycle-related proteins in gallbladder cancer cells.

To determine the effect of cordycepin on the cell cycle distribution of gallbladder cancer cells, we performed flow cytometry assays. As shown in Figure 3A–D, cordycepin significantly decreased the number of cells in the G_0_/G_1_ phase and significantly increased the percentage of NOZ and GBD-SD cells in the S phase. These results indicate that cordycepin arrests the cell cycle at the S phase in a dose-dependent manner and can suppress tumor growth by preventing proper DNA replication.

Additionally, we investigated the effects of cordycepin on cell cycle-related proteins. After treatment with cordycepin for 48 h, the expression of the cell cycle regulatory proteins, cyclin A and Cdk-2, which represent two key regulators of the S phase [22], was decreased in a concentration-dependent manner (Figure 3E,F). This result suggests that cordycepin induces cell cycle arrest by regulating S phase-related proteins in gallbladder cancer cells. Moreover, the cyclin inhibition protein 1 (P21) and kinase inhibition protein 1 (P27), which can inhibit the CDK kinase activity [23,24], were upregulated. All of these further conformed that cordycepin can induce S phase arrest in human gallbladder cancer cells.

(**A**,**B**) NOZ and GBC-SD cells were treated with various concentrations of cordycepin for 48 h, and the DNA content was analyzed by flow cytometry. (**C**,**D**) The percentages of cells in the G1, S and G2/M phases of the cell cycle are shown. Results are represented as the mean ± SD from three independent trials. (**E**,**F**) The effects of cordycepin on the protein levels of cyclin A, Cdk-2, P21 and p27 in NOZ and GBC-SD cells were investigated by western blot analysis after treatment with different concentrations of cordycepin for 48 h. β-actin was used as a loading control.

### 2.3. Cordycepin Induces Apoptosis in Human Gallbladder Cancer Cells

**Figure 4:**
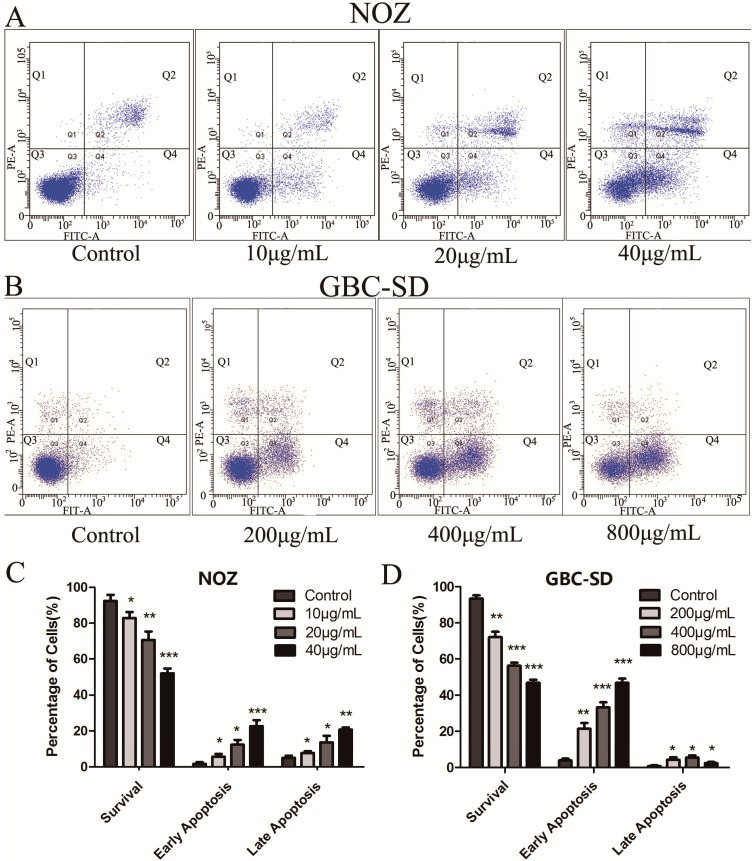
Cordycepin induces the apoptosis of human gallbladder cancer cells.

To further evaluate the apoptosis-inducing capability of cordycepin in NOZ and GBC-SD cells, Annexin V-FITC/PI double staining and flow cytometry were conducted. Annexin V-FITC positivity and PI negativity (Q4 quadrant) were considered to represent early apoptotic cells; Annexin V-FITC positivity and PI-positivity (Q2 quadrant) represented late apoptotic cells; and Annexin V-FITC negativity and PI negativity (Q3 quadrant) indicated non-apoptotic cells. After treatment with cordycepin for 48 h, the number of viable cells was reduced, whereas the numbers of both early and late apoptotic cells were significantly increased in a dose-dependent manner. As shown in Figure 4A–D, treatment with 40 μg/mL cordycepin induced early apoptosis in approximately 22.6% ± 3.40% and late apoptosis in approximately 20.7% ± 1.21% of NOZ cells. In addition, treatment with 800 μg/mL cordycepin induced early apoptosis in approximately 46.7% ± 2.38% and late apoptosis in approximately 2.4% ± 0.70% of GBC-SD cells. These data indicate that cordycepin may inhibit the proliferation of gallbladder cancer cells through an apoptosis pathway.

(**A**,**B**) NOZ and GBC-SD cells were exposed to different concentrations of cordycepin for 48 h, followed by staining with Annexin-V/PI. The Q3 quadrant (Annexin V−/PI−), Q4 quadrant (Annexin V+/PI−) and Q2 quadrant (Annexin V+/PI+) represent the percentage of non-apoptotic cells, early apoptotic and late apoptotic cells, respectively. (**C**,**D**) Compared with the control group, the ratios of non-apoptotic cells, early apoptotic cells and late apoptotic cells are shown. The data represent the mean ± SD of three independent experiments. * *p* < 0.05, ** *p* < 0.01, *** *p* < 0.001 compared with the control.

### 2.4. Cordycepin Reduces ΔΨm in Human Gallbladder Cancer Cells

**Figure 5:**
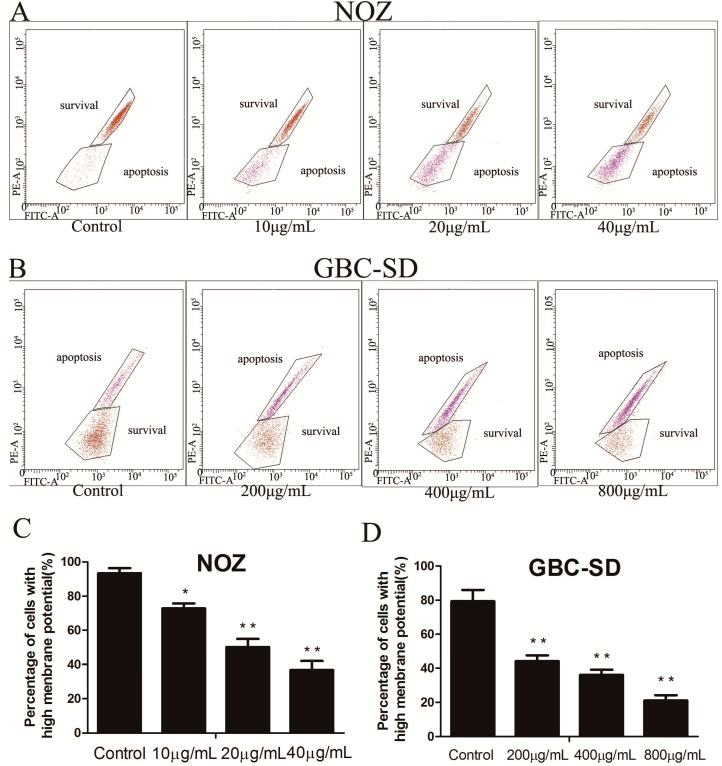
Cordycepin reduces ΔΨm in human gallbladder cancer cells.

A variety of key events during apoptosis involve the mitochondria. Moreover, cells cannot gain a growth advantage simply by losing mitochondria, and mitochondria dysfunction is often associated with changes in ΔΨm. To evaluate whether mitochondrial membrane integrity was damaged by treatment with cordycepin, ΔΨm in human gallbladder cells was examined using Rhodamine 123, a yellow-green fluorescent probe that stains mitochondria in living cells in a membrane potential-dependent manner. In Figure 5A–D, purple and brown represent apoptosis and survival respectively, and the results show that cordycepin induced a dose-dependent decrease in ΔΨm. In particular, we found that more than 65% of NOZ cells and 78% of GBC-SD cells showed a reduction in ΔΨm after treatment with cordycepin (40 μg/mL for NOZ and 800 μg/mL for GBC-SD) for 48 h. These data indicate that cordycepin induces apoptosis in human gallbladder cancer cells through the mitochondria-related pathway. In this figure, the survival and apoptosis area were opposite in NOZ and GBC-SD cells; this conclusion was drawn through the peak value of each cell line’s fluorescence intensity in the flow cytometric analysis.

### 2.5. Cordycepin Induces Apoptosis by Regulating Bcl-2 Family Members and Caspase-3 in Human Gallbladder Cancer Cells

**Figure 6:**
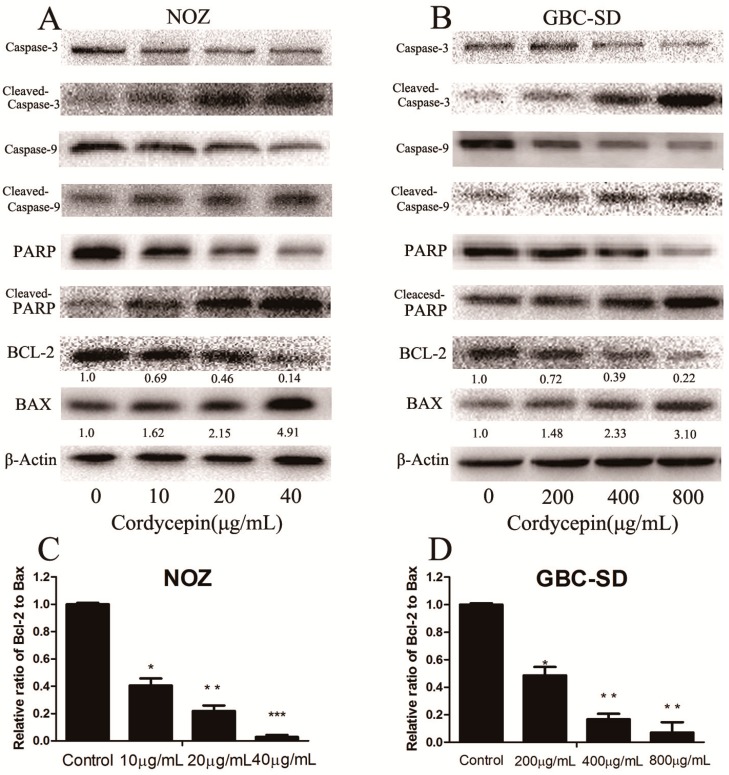
Cordycepin regulates the expression of apoptosis-related proteins in human gallbladder cancer cells.

To evaluate the molecular mechanism responsible for the apoptotic effect of cordycepin on NOZ and GBC-SD cells, the expression of apoptosis-related proteins (caspase-3, cleaved-caspase-3, caspase-9, cleaved-caspase-9, PARP, cleaved-PARP, Bax and Bcl-2) was evaluated by western blot analysis after treatment with various concentrations of cordycepin for 48 h. As shown in Figure 6A,B, Bcl-2 and the total form of caspase-3, caspase-9 and PARP expression were downregulated, whereas Bax, cleaved caspase-3 and cleaved PARP were upregulated in a dose-dependent manner. We also measured the Bax/Bcl-2 ratio at the protein level (Figure 6C,D), which was significantly increased in the cordycepin-treated groups compared with the control group. Because these molecules play important roles in apoptosis, they may be responsible for the cordycepin-induced apoptosis observed in NOZ and GBC-SD cells.

The deregulation of apoptosis serves as an indicator of carcinogenesis [25], and the induction of apoptosis is a standard strategy used in anticancer therapy [26,27]. Apoptosis can be initiated via two major pathways: the mitochondria-mediated intrinsic pathway and the death receptor-induced extrinsic pathway, both of which ultimately activate effector caspases and apoptosis effector molecules [28,29]. *Cordyceps sinensis*, which is widely used in traditional Chinese medicine, had been shown to possess anticancer effects in a broad range of human cancer cells by regulating the cell cycle and apoptosis [30,31,32]. However, there have been no reports about the effect of cordycepin on human gallbladder cancer. In the present study, we investigated the potential mechanism of apoptosis induced by cordycepin and found that the mitochondria-medicated intrinsic pathway played an important role in cordycepin-mediated apoptosis. After treatment with cordycepin for 48 h, both NOZ and GBC-SD cells underwent a decrease in ΔΨm, suggesting that cordycepin induced apoptosis in gallbladder cells. Moreover, the Bcl-2 gene family, which includes some of the best-studied anti-apoptotic factors, is the key regulator of the mitochondria-mediated pathway [33]. This pathway contains several members, including Bax, Bcl-2 and Bid, among which the anti-apoptotic protein, Bcl-2, and the apoptosis-promoting protein, Bax, play a leading role in regulating apoptosis [34,35]. The ratio of Bcl-2/Bax is also used to evaluate the occurrence and severity of apoptosis, and caspase apoptosis proteins are activated when this ratio is reduced [36]. We found that the Bcl-2/Bax ratio decreased by 34.4- and 14.1-fold compared with the control group after treatment with cordycepin (40 μg/mL for NOZ cells and 800 μg/mL for GBC-SD cells) for 48 h, as assessed by western blot assay. These data suggest that the decrease in the Bcl-2/Bax ratio is correlated with the apoptosis induced in human gallbladder cancer cells by cordycepin.

(**A**,**B**) Flow cytometric analysis of ΔΨm via Rhodamine 123 staining. Survival indicated cells with high ΔΨm, and apoptosis indicated cells with low ΔΨm. Purple and brown represent apoptosis and survival, respectively. (**C**,**D**) The percentage (%) of cells with high ΔΨm. Values are presented as the mean ± SD of three independent trials. * *p* < 0.05; ** *p* < 0.01.

(**A**,**B**) The expression of caspase-3, caspase-9, PARP, cleaved-caspase-3, cleaved-caspase-9, cleaved-PARP, Bax and Bcl-2 in NOZ and GBC-SD cells was investigated by western blot analysis after treatment with various concentrations of cordycepin for 48 h. β-actin was used as a loading control. (**C**,**D**) The Bcl-2/Bax ratio was evaluated by the band density compared with the control (designated as 1.00), and the results are presented as the mean ± SD. * *p* < 0.05; ** *p* < 0.01; *** *p* < 0.001.

Our study also investigated the caspase family, which consists of cysteine proteases that are indispensable in the execution process of apoptosis. Caspase-9 is activated in the mitochondria-mediated intrinsic pathway, and caspases-3 is a key regulator in the caspase-dependent cell apoptosis pathway. In addition, the activation of caspase-3 leads to the final destruction of a target cell [37]. A number of cellular proteins, such as PARP, are cleaved following the activation of caspases. We observed that caspase-3 and caspase-9 were both activated after treatment with cordycepin for 48 h in human gallbladder cells, which was accompanied by the increased cleavage of PARP. This revealed that the involvement of a caspase-dependent pathway through a caspase-9-triggered mitochondrial pathway may lead to cordycepin-induced apoptosis.

### 2.6. Cordycepin Suppresses Tumor Growth in Vivo

**Figure 7:**
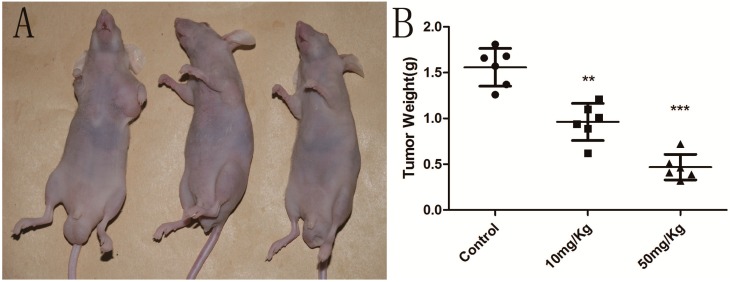
Cordycepin suppress tumor growth *in vivo*.

It was reported that cordycepin has no effect on bodyweight loss or systemic toxicity through intragastric or intraperitoneal administration at the maximal tolerant dose in mice [38,39]. Therefore, we further investigated whether cordycepin could decrease tumor growth *in vivo*, as treatment with cordycepin was shown to suppress the growth of human gallbladder cells *in vitro*. Our results indicated that the tumor growth in nude mice injected with NOZ tumor cells was significantly reduced in a dose-dependent manner without any loss of bodyweight or systemic toxicity after treatment with cordycepin compared with mice injected with PBS vehicle alone (Figure 7) (*p* < 0.05). Thus, our results show that cordycepin could effectively suppress tumor growth *in vivo*.

(**A**) Cordycepin induced the suppression of tumor growth in nude mice injected with NOZ cells. One representative mouse from each group is shown. (**B**) Tumors were weighed in each group. The data are illustrated as the mean ± SD (n = 6). ** *p* < 0.01, *** *p* < 0.001.

## 3. Experimental Section

### 3.1. Drugs and Antibodies

William’s medium, Dulbecco’s Modified Eagle’s Medium (DMEM), antibiotics, fetal bovine serum and trypsin were purchased from Gibco (Carlsbad, CA, USA). Cordycepin was obtained from the National Institute for the Control of Pharmaceutical and Biological Products (Beijing, China). An Annexin V/Dead Cell Apoptosis Kit was purchased from Invitrogen (Carlsbad, CA, USA). The primary antibodies used for western blotting were as follows: rabbit anti-Bcl-2, anti-Bax, anti-caspase-3, anti-caspase-9, anti-PARP, anti-cleaved-caspase-3, anti-cleaved-caspase-9, anti-cleaved-PARP, anti-Cdk-2, anti-Cyclin A and mouse anti-β-actin. All of the antibodies were purchased from Cell Signaling Technology (Beverly, MA, USA).

### 3.2. Cell Lines and Culture

The human gallbladder cancer cell line NOZ was purchased from the Cell Bank of Type Culture Collection of the Chinese Academy of Sciences (Shanghai, China). The human gallbladder cancer cell line GBC-SD was purchased from the Shanghai Institute of Cell Biology, Chinese Academy of Sciences (CAS). NOZ cells were cultured in William’s medium (Gibco, Grand Island, NY, USA) supplemented with 100 U/mL penicillin-streptomycin (Hyclone, Logan, UT, USA) and 10% fetal bovine serum (Gibco, Grand Island, NY, USA) in a humidified incubator at 37 °C and 5% CO_2_. GBC-SD cells were cultured in high-glucose DMEM (Gibco, Grand Island, NY, USA) supplemented with 100 U/mL penicillin-streptomycin and 10% fetal bovine serum at 37 °C in an incubator containing 5% CO_2_.

### 3.3. Cell Proliferation Assay

NOZ and GBC-SD cells were seeded into 96-well plates at a density of 4 × 10^3^ cells per well and incubated overnight. Then, the cells were treated with cordycepin at different concentrations (0, 5, 10, 20, 40 and 60 μg/mL for NOZ cells and 0, 0.05, 0.1, 0.2, 0.4 and 0.8 mg/mL for GBC-SD cells). Cell viability was quantified using a Cell Counting Kit-8 (CCK-8, Dojindo, Kumamoto, Japan) at 24, 48 and 72 h after culture with cordycepin, as previously described. The optical density was measured at 450 nm using a microtiter plate reader (Quant Bio Tek Instruments, Winooski, VT, USA), and the cell survival rate was expressed as the absorbance relative to that of untreated controls. The results represent the average of three independent experiments conducted over multiple days.

### 3.4. Colony Formation Assay

Cells in a logarithmic growth phase were trypsinized into single-cell suspensions and plated in a 6-well plate at a density of 400 cells per well. After adherence, NOZ and GBC-SD cells were treated with cordycepin for 48 h (0, 2, 4 and 6 μg/mL for NOZ; 0, 20, 40 and 80 μg/mL for GBC-SD). Cells were cultured for approximately 14 days, fixed with 4% paraformaldehyde and stained with 0.1% crystal violet (Sigma-Aldrich, St. Louis, MO, USA). After washing, plates were air-dried, and stained colonies were photographed using a microscope (Leica, German). The total number of colonies (>50 cells/colony) was counted, and the results are represented as the average of three independent experiments conducted over multiple days.

### 3.5. Cell Cycle Analysis

NOZ and GBC-SD cells were treated with various concentrations of cordycepin (0, 10, 20 and 40 μg/mL for NOZ cells and 0, 0.2, 0.4 and 0.8 mg/mL for GBC-SD cells) for 48 h. Then, the cells were collected and washed twice with PBS. After fixing the cells in cold 70% ethanol at 4 °C overnight, the cells were washed and then resuspended in cold PBS and incubated with 10 mg/mL RNase and 1 mg/mL propidium iodide (PI) (Sigma-Aldrich, St.Louis, MO,USA) at 37 °C for 30 min in the dark. The samples were analyzed by flow cytometry (BD Biosciences, San Diego, CA, USA), and the percentage of cells in the G_0_/G_1_, S and G_2_/M phases was determined using Cell Quest acquisition software (BD Biosciences, San Diego, CA, USA).

### 3.6. Cell Apoptosis Assay

The analysis of apoptosis was performed according to the manufacturer’s instructions for the Annexin V/PI apoptosis kit (Invitrogen, Carlsbad, CA, USA). NOZ and GBC-SD cells were seeded in 6-well plates (Corning, Corning, NY, USA) with 1 × 10^6^ cells per well and treated with different concentrations of cordycepin (0, 10, 20 and 40 μg/mL for NOZ cells and 0, 0.2, 0.4 and 0.8 mg/mL for GBC-SD cells) for 48 h. Then, the cells were harvested and washed twice with cold PBS and resuspended at a density of 1 × 10^6^ cells/mL in 100 µL of binding buffer containing 5 µL of Annexin V-FITC and 1 μL of PI working solution (100 μg/mL). After incubation at room temperature for 15 min in the dark, 400 μL of binding buffer were added to each sample. Apoptosis was analyzed by flow cytometry (BD, San Diego, CA, USA) for at least 10,000 events.

### 3.7. Mitochondrial Membrane Potential (ΔΨm) Assay

The ΔΨm was analyzed by fluorescence microscopy using the Rhodamine 123 (Sigma-Aldrich, St. Louis, MO, USA) probe. After treatment with various concentrations of cordycepin (0, 10, 20 and 40 μg/mL for NOZ cells and 0, 0.2, 0.4 and 0.8 mg/mL for GBC-SD cells) for 48 h, the cells were collected and washed twice with cold PBS, followed by incubation with Rhodamine 123 at 37 °C for 30 min in the dark. After that, the cells were washed twice with cold PBS and analyzed by flow cytometry.

### 3.8. Western Blot Analysis

After treatment with cordycepin (0, 10, 20 and 40 μg/mL for NOZ cells and 0, 0.2, 0.4 and 0.8 mg/mL for GBC-SD cells) for 48 h, the cells were collected and lysed in RIPA buffer (Beyotime, Shanghai, China) supplemented with protease inhibitor (Roche Applied Science, Indianapolis, IN, USA). The total protein concentration of each sample was determined using the BCA protein assay (Beyotime, Shanghai, China) with BSA as a standard. Equal protein extracts (60 μg protein per lane) were separated by SDS-PAGE and electrophoretically transferred to polyvinylidene difluoride membranes (Millipore, Bedford, MA, USA). Then, the membranes were blocked with 5% skim milk and incubated with anti-caspase-3, anti-caspase-9, anti-Bcl-2, anti-Bax, anti-PARP, anti-Cyclin A, anti-CDK2, anti-21, anti-p27 and anti-β-actin antibodies (1:1,000; Cell Signaling Technology) at 4 °C overnight, followed by incubation with a horseradish peroxidase-conjugated goat anti-rabbit/anti-mouse secondary antibody (1:5,000; Abcam, Cambridge, UK). Proteins were visualized using the Gel Doc 2000 (BioRad, Hercules, CA, USA).

### 3.9. In Vivo Efficacy of Cordycepin

Six- to eight-week-old BALB/c homozygous (nu/nu) nude mice (18–20 g body weight) were purchased from Shanghai SLAC Laboratory Animal Co., Ltd. (Shanghai, China). The mice were maintained in a specific pathogen-free environment. All procedures were approved by the Institutional Animal Care and Use Committee of Shanghai Jiao Tong University and were carried out in accordance with the institutional guidelines of Shanghai Jiaotong University (Shanghai, China). NOZ cells (2.5 × 10^6^) in log-phase growth were resuspended in 100 μL PBS and then injected into the left axilla of nude mice. On Day 5, these mice were randomly divided into three groups (6 mice/group). The first group received an intraperitoneally (*i.p.*) injection of William’s medium each day. The other two groups were administered an *i.p.* injection of cordycepin at 10 mg/kg and 50 mg/kg every day. On Day 22, all nude mice were sacrificed, and the tumor tissue was removed and weighed.

### 3.10. Statistical Analysis

Statistical analyses were conducted using SPSS 18.0 software. All assays were performed independently three times, and all quantified data are expressed as the mean ± SD or as indicated. Statistical significance was calculated using the Student’s *t*-test, and p-values of less than 0.05 (* *p* < 0.05, ** *p* < 0.01, *** *p* < 0.001) were considered statistically significant for all tests.

## 4. Conclusion

Our study revealed that cordycepin suppressed the proliferation and induced the apoptosis of NOZ and GBC-SD cells via the activation of the mitochondrial-mediated intrinsic caspase pathway. These results indicate that cordycepin can have a significant anti-tumor effect in human gallbladder cancer cells, which suggests the potential application of cordycepin in the development of new anticancer drugs for the treatment of gallbladder cancer.
